# Drug Induced Hypersensitivity and the HLA Complex

**DOI:** 10.3390/ph4010069

**Published:** 2010-12-23

**Authors:** Ana Alfirevic, Munir Pirmohamed

**Affiliations:** The Wolfson Centre for Personalised Medicine, Department of Pharmacology and Therapeutics, University of Liverpool, Block A: Waterhouse Buildings, 1-5 Brownlow Street, Liverpool L69 3GL, UK

**Keywords:** drug-induced hypersensitivity, pharmacogenetics, human leukocyte antigen (HLA), major histocompatibility complex, predictive genetic testing

## Abstract

Drug-induced hypersensitivity reactions are of major concern and present a burden for national healthcare systems due to their often severe nature, high rate of hospital admissions and high mortality. They manifest with a wide range of symptoms and signs, and can be initiated by a wide range of structurally diverse chemical compounds. The pathophysiological mechanisms underlying hypersensitivity reactions are not well understood, but it is thought that they are immune mediated. MHC region on Chromosome 6 contains many genes with immune function. Classical MHC molecules are highly polymorphic cell surface glycoproteins whose function is to present peptide antigens to T cells. In addition to conferring protection from some diseases, HLA alleles are also associated with an increased risk of other diseases, including drug-induced hypersensitivity. Pharmacogenetic approach to predict the risk of drug-induced hypersensitivity has been established for several drugs. We will discuss the progress of hypersensitivity pharmacogenetics over the last few years and focus on current efforts of the international community to develop consortia which aim to standardize disease phenotypes and to identify affected individuals through international collaborations. In addition, we will discuss the clinical utility of HLA typing as predictive or diagnostic testing for drug-induced hypersensitivity.

## 1. Definitions

### 1.1. Hypersensitivity

Drug-induced hypersensitivity reactions represent a heterogeneous group of Type B or “off target” adverse drug reactions (ADRs), which manifest with a wide range of clinical symptoms and signs, and can be initiated by a wide range of structurally diverse chemical compounds. Hypersensitivity reactions are of major concern and present a burden for national healthcare systems due to their often severe nature, high rate of hospital admissions and high mortality [1-3]. The pathophysiological mechanisms underlying hypersensitivity reactions are not well understood, but general agreement among clinicians and researchers is that they are immune mediated [4,5]. One of the most commonly reported reactions is delayed type hypersensitivity, which is T cell mediated.

### 1.2. HLA Alleles

The major histocompatibility complex (MHC), which spans approximately 3.6 Mb on band 6p21.3 of the short arm of chromosome 6 has an essential role in the innate and adaptive immune system. It contains a large number of highly polymorphic genes characterised by high linkage disequilibrium. Because of the importance of the MHC region in immunity, and in the aetiology of many autoimmune diseases, great effort has been made to sequence, analyse and annotate this region [6,7]. The highest quality sequence has been achieved by manual annotation (VEGA database http://vega.sanger.ac.uk/). The MHC region includes genes in the human leukocyte antigen (HLA) Class I, II and III. HLA class I contains HLA-A, HLA-B and HLA-C, Class II contains HLA-DR, HLA-DP and HLA-DQ, while Class III contains various genes which have immune related functions including the tumour necrosis factor alpha (TNF) gene, lymphotoxin alpha (LTA), heat shock proteins and many other non-immune related genes (please see http://hla.alleles.org/nomenclature/stats.html). The nomenclature of HLA alleles has been updated recently [8].

Classical MHC molecules are highly polymorphic cell surface glycoproteins whose function is to present peptide antigens to T cells. Antigen presentation is crucial for the regulation of protective immune responses against pathogens and for the maintenance of self-tolerance. The huge diversity in peptide binding is driven by the diversity and mutations in infectious agents that the human race has encountered through evolution. MHC polymorphisms are needed to maximize peptide binding; each variant of a given MHC molecule can bind different peptides. It is thought that clustering of the genes encoding the MHC molecules may increase recombination which generates new polymorphisms. HLA-B is the most polymorphic gene in the human genome; it contains more than 1,600 alleles (http://www.ebi.ac.uk/imgt/hla/). Given that each individual inherits one paternal and one maternal chromosome and a large number of available alleles for each of the HLA Class I alleles (HLA-A, -B and -C), it is unlikely that two individuals will have the same six MHC Class I molecules. HLA alleles may also confer protection against infectious and immune diseases. In such instances, it is advantageous for an individual to be heterozygous for each MHC molecule. An example of heterozygous advantage is resistance to HIV in Caucasian individuals possessing HLA-B*5701 [9], or B*5703 in African Americans [10]. Interestingly however, alleles strongly associated with HIV-1 disease progression showed no effect in HIV-2 disease [11]. The effects of HLA combinations on HIV-2 immune responses relative to HIV-1 could be related to their distinct clinical course; unlike those infected with HIV-1virus, many individuals infected with HIV-2 virus remain healthy throughout their life.

In addition to conferring protection from some diseases, HLA alleles are also associated with an increased risk of other diseases, in particular, autoimmune diseases and drug-induced hypersensitivity. The latter is the main topic of this article and is going to be reviewed in some detail. In addition, we will discuss the clinical utility of HLA typing as predictive or diagnostic testing for drug-induced hypersensitivity.

## 2. Diagnosis

### 2.1. Clinical Manifestations

Delayed type hypersensitivity reactions can be induced by a large number of drugs. They can occur a few hours or up to several weeks after drug intake, and therefore causality is sometimes difficult to establish based on time-event relationships. The clinical manifestations vary from mild skin rashes which do not require any therapeutic intervention, to the other end of the spectrum, where skin reactions can be accompanied by systemic symptoms and multiple organ involvement that can be life-threatening (please see Table 1). However, given the heterogeneity of clinical symptoms [12], difficulties in obtaining a reliable clinical history and lack of reliable and simple diagnostic tests, it is often very difficult to diagnose drug-induced hypersensitivity.

As a consequence of this, a large variety of phenotypic definitions and related assessment criteria exist in the published literature. An international initiative to standardise clinical phenotype nomenclature has thus recently been started through the Phenotype Standardization Project (PSP) (http://www.saeconsortium.org/?q=node/23/). The PSP has been organized by the US FDA, the International Serious Adverse Events Consortium (iSAEC) and the Wellcome Trust with the main aim to develop standardized phenotypes, which could facilitate Electronic Medical Record (EMR) identification of potential cases.

### 2.2. Testing for Drug-Induced Hypersensitivity

*In vivo* and *in vitro* tests are available to confirm the diagnosis of hypersensitivity; however, their clinical utility has been rather low. For instance, skin patch testing has been reviewed recently in relation to anticonvulsant hypersensitivity syndrome [13]. The patch test is an *in vivo* immune function test used to measure the presence of activated T cells that recognize medications that caused delayed hypersensitivity reactions. T cell responses occur over several days and lead to induration (hardening) and erythema of the skin over the area exposed to suspect drug. Skin patch testing can be used to immunologically confirm suspected cases of hypersensitivity, as has been done with the antiretroviral drug abacavir [14] Abacavir hypersensitivity reactions can be particularly difficult to diagnose because they may mimic the infections that frequently occur in HIV patients. In addition, given the number of medications that HIV patients receive, it may be difficult to establish the culprit drug because the symptoms from different drugs are often similar. It is however important to note the recent FDA warning that skin patch testing with a drug that caused hypersensitivity can lead to fatal reactions, and the use of such a test should be based on a careful consideration of the risk-benefit relationship (http://www.accessdata.fda.gov/scripts/cdrh/cfdocs/psn/transcript.cfm?show=84#3). Arguably, the main reason preventing wider adoption of the skin patch test is its unknown sensitivity and specificity. Its positive predictive value seems to be higher than its negative predictive value, and therefore another test should be used to confirm the diagnosis in case of a negative patch test. Systemic re-challenge may be an option however, but should not be used in a patient with severe hypersensitivity reactions, for example in DRESS or SJS/TEN. Furthermore it is important to note that current guidelines often recommend discontinuing suspected medications in patients who develop rash.

*In vitro* diagnostic tests have the clear advantage over *in vivo* tests in that they are safe to use. Two of these, the lymphocyte transformation test (LTT) [15] and lymphocyte toxicity assay (LTA) [16] have been successfully employed in specialised laboratories as research tools, but have not been translated into clinical practice [17]. Both methods utilize peripheral blood mononuclear cells (PBMCs) as target cells. The LTT assay entails the measurement of T-cell proliferation *in vitro*: The theory is that peripheral lymphocytes proliferate from a drug-hypersensitive patient when incubated *in vitro* with the culprit drug at non-toxic concentrations. The rate of cell proliferation can be measured in a number of ways, the most common being [^3^H]-thymidine incorporation which is expressed as a ratio between proliferation of cells incubated with and without the drug (vehicle alone; control) and termed the stimulation index.

LTA, on the other hand, is an *in vitro* test that utilizes isolated PBMCs to investigate the mechanistic pathogenesis of idiosyncratic drug reactions. The test is based on the hypothesis that drug hypersensitivity develops as a consequence of the formation of toxic reactive metabolites together with a lack of detoxification capacity in PBMCs which serve as a surrogate for other cells in the body [16]. In this test, PBMCs isolated from the patient are incubated with the culprit drug in the presence of induced or un-induced liver microsomes (mouse, rat, rabbit or human), which are a source of cytochrome P_450_ mono-oxygenase activity. The percentage cell death is then assessed using trypan blue exclusion or tetrazolium dye method. The cell death allegedly reflects the sensitivity of the cells to the toxic effects of the culprit drug in patients with hypersensitivity compared to tolerant patients. However, although this test has been used in a number of laboratories, the mechanistic basis of the increase in cell death in the sensitive patients has never been fully explained. Furthermore, the test is labour-intensive, and its true sensitivity, specificity, positive and negative predictive values have never been fully elucidated.

## 3. Approaches to the Genetic Investigation of Drug Hypersensitivity

Our lack of understanding of the underlying mechanisms of drug hypersensitivity, together with the small number of hypersensitive patients available for study for each individual drug, has led to different approaches in investigating the genes responsible for these serious adverse drug reactions. Most of the investigations have utilised a candidate gene approach based on the known metabolic pathways of the implicated drugs, and because of the presumed immune aetiology, also investigated the HLA and cytokine genes. An alternative approach described by Yang involved the *in silico* investigation of the chemical-protein interactome. The authors interrogated more than 160 drugs known to cause SADRs and more than 840 protein targets [18] and analysed drug-protein binding strength and binding conformation. Interestingly, the data-mining strategies revealed and predicted the binding interactions that mediate hypersensitivity; for example, abacavir was found to bind to the antigen presentation groove of MHC I molecule in patients carrying the B*5701 allele but not B*5703. The authors further developed the hypothesis that drugs with similar phenotypic effects interact with same target proteins and that the key interacting residues and the risk alleles for each individual could be identified *in silico*. Despite the promising results, the utility of such *in silico* approaches needs to be determined, as early adoption could potentially lead to abandonment of new drugs which in practice may not have caused hypersensitivity. In another study, the genetic control of antigen recognition was explored by Archbold and colleagues [19]. They showed how different HLA-B*44 allotypes (B*4402, B*4403 and B*4405) show marked differences in the epitope region and consequently their affinity to and recognition by the T-cell receptor. The importance of this in relation to drug hypersensitivity has been confirmed by the findings of recent studies which have shown the necessity of 4-digit HLA genotyping (discussed with respect to individual drugs below).

Rapid progress in genotyping technologies over the past few years has led to increasing utilisation of genome-wide association study (GWAS) approaches. Over 665 studies had been published by the end of September 2010 and a GWAS catalogue is available at http://www.genome.gov/gwastudies [20]. One of many advantages of the GWAS approach is its unbiased nature, which enables identification of novel susceptibility factors. The GWAS approach is hypothesis generating and data driven, rather than hypothesis testing. It can simultaneously interrogate up to 2 million SNPs, providing that the appropriate study design is applied. Of all the GWAS studies performed to date, only a small subset relates to the genetics of drug-response. Similar to all pharmacogenetic studies, the success of any GWAS will depend on several factors including the effect size and allele frequency of genetic variants responsible for the relevant phenotype, the sample size, the population under study and study design [21]. A large sample size is particularly difficult to achieve in drug-induced hypersensitivity studies given the low incidence of serious reactions and the difficulties sometimes encountered in defining an accurate phenotype. Although there is a clear advantage of using GWAS to study hypersensitivity, it is also important to remember that very few novel *loci* outside the MHC have been discovered to date. Clearly, the HLA genes are the usual candidates for drug hypersensitivity, and as such careful study here is likely to lead to significant findings, as for example was demonstrated for abacavir hypersensitivity and HLA-B*5701 [22-24].

## 4. Risk Factors

### 4.1. Viral Infections

An interaction between viral infections and drug-induced hypersensitivity has been most often associated with ampicillin-induced exanthema in patients with infectious mononucleosis caused by Epstein Barr virus (EBV) [25]. Exanthematous eruptions occur in approximately 10% of patients with infectious mononucleosis, but this rate can increase to 70% in adults and 100% in children receiving ampicillin. Currently, there is on-going debate as to whether this is true hypersensitivity [26]; the LTT assay has however helped to demonstrate the immune aetiology of the disease [27,28].

Another well known example of a relationship between viral infection and an increased risk of developing drug-induced skin rashes, including SJS and TEN, has been observed in HIV-positive patients [29]. Clinical observations and several studies showed that in the pre-HAART era, the incidence of severe adverse reactions to drugs such as co-trimoxazole was much higher in HIV patients than in the general population [30].

Viral infections have been suggested as a potential trigger for hypersensitivity reactions. This is particularly the case with HHV-6 infection and anticonvulsant-induced hypersensitivity [25,31]. It has been suggested that since HHV-6 reactivation can only be detected in hypersensitivity syndrome and not in other drug reactions, it can be utilised as a diagnostic test for hypersensitivity. Indeed, in Japan, HHV-6 reactivation seems to be a gold standard test for drug-induced hypersensitivity syndrome [32]. In addition, slow resolution of DRESS is thought to be linked to HHV-6 reactivation and hypogammaglobulinaemia which can occur during treatment with certain drugs, in particular anticonvulsants [31]. The herpes group family of DNA viruses including EBV, CMV, HHV-6, HHV-7 and HSV, have not only been implicated in drug-induced hypersensitivity reactions but also in SJS, where viral DNA has been identified in the blood of patients [33]. These viruses are important opportunistic pathogens, which can induce massive expansions of cross reactive memory T-cells [25]. The demonstrated relationships between viral infections and hypersensitivity is presented in Table 2.

### 4.2. Ethnicity

Different HLA alleles seem to represent important risk factors for hypersensitivity induced by several drugs in patients of variable genetic ancestry. The most commonly discussed example is carbamazepine-induced SJS/TEN. While a strong association has been demonstrated between HLA-B*1502 and SJS/TEN in Han Chinese and Thai populations, no such relationship has been seen in Caucasians or Japanese individuals presumably because of the relative rarity of the B*1502 allele in these patient groups [34-38]. In contrast to carbamazepine, abacavir hypersensitivity is associated with HLA-B*5701 in patients of all ethnic backgrounds, including Caucasians and Black Africans [39]. Mechanistically, *in vitro* studies have suggested that B*5701 allele is the causative variant [40], but in contrast, mechanistic evidence that HLA-B*1502 is the causative allele for CBZ-induced SJS/TEN is still lacking [41]. Table 3 presents an overview of the role of ethnicity and the corresponding HLA alleles in drug hypersensitivity.

### 4.3. Genetics

Observational family studies, as well as identical twin studies, have shown that there is a genetic component to hypersensitivity reactions. Genetic susceptibility factors, in particular HLA alleles, have been identified as risk factors for hypersensitivity since the 1980s. For instance, several markers within the MHC region have been associated with hypersensitivity to the antimicrobials, sulphamethoxazole and penicillin, and to non-steroidal antiinflammatory drugs including oxicams (listed in Table 2). However, the low predictive values have prevented them from being used for the prediction or prevention of ADRs in clinical practice. The first major breakthrough in searching for genetic markers associated with drug-induced hypersensitivity came in 2002 with two studies on abacavir hypersensitivity showing an association with HLA-B*5701 [22,24]. Several other well defined examples include serious cutaneous adverse reactions to carbamazepine and allopurinol, and drug-induced liver injury associated with a number of drugs, presumed to be immune-mediated. These examples will be discussed in more detail.

## 5. Antiretrovirals

### 5.1. Abacavir Hypersensitivity and HLA-B*5701

Abacavir is a nucleoside reverse transcriptase inhibitor used as an anti-HIV agent. Approximately 5-9% of patients develop abacavir hypersensitivity (AHS) [42]. It is thought that the reaction is CD8^+^ T-cell mediated as evidenced in skin biopsies taken from patients with positive patch tests to abacavir [43]. The association between the HLA Class I allele HLA-B*5701 and AHS was reported by two groups with a high odds ratio [22,24]. Since then, several other groups have demonstrated that: (1) abacavir HLA-B*5701 pre-treatment testing is cost effective [23,44]; (2) B*5701 is useful genetic marker for prediction of hypersensitivity in several ethnic groups including the white and black African ancestry [39]; (3) a simple test for the detection of B*5701 allele is available through the HCP5 SNP rs2395029 which is in almost complete (although not total) LD with HLA-B*5701 [45]; (4) the clinical utility of pre-treatment B*5701 testing has been shown in a prospective randomised controlled clinical trial [14]; and (5) *in vitro* studies have demonstrated that abacavir treated antigen presenting cells positive for HLA-B*5701, but not the related alleles B*5702 or B*5801, can stimulate abacavir specific CD8^+^ T cell responses [40]. The latter provides evidence of the functional relevance of B*5701 in the mechanism of AHS. All of these factors, taken together with the fact that HIV physicians were amenable to change, and there was a vocal patient lobby, contributed to the successful implementation of abacavir pre-treatment genetic testing in high-income countries. A high negative predictive value but a relatively low positive predictive value (48%) characterises the test [14]. Therefore nearly 50% of HLA-B*5701 positive individuals will not develop hypersensitivity in response to abacavir, and thus, other genetic or non-genetic factors may play a role in abacavir hypersensitivity.

### 5.2. Nevirapine

Nevirapine, a non-nucleoside reverse transcriptase inhibitor, is used in combination with other antiretrovirals as part of Highly Active Antiretroviral Therapy (HAART). Nevirapine can cause hypersensitivity reactions or isolated liver toxicity often characterised by jaundice [46,47]. Unlike abacavir-induced hypersensitivity, the HLA association for nevirapine hypersensitivity has not been as clear-cut. The genetic basis for nevirapine hypersensitivity has been supported by case reports in members of the same family [48]. However, it seems that the immunogenetic pathogenesis is more complex than that of abacavir in that both MHC Class I and Class II alleles have been implicated in different populations [49-51]. While a Western Australian and a French study showed an association between nevirapine hypersensitivity and the MHC Class II allele HLA-DRB1*0101 [51,52], two further studies suggested the involvement of MHC Class I alleles HLA-Cw8/HLA-B14 [50] and HLA-Cw8 [49] in Sardinian and in Japanese populations, respectively. To add to the complexity, in two separate studies in the Thai population it was HLA-Cw4 and HLA-B*3505 that were found to be associated with nevirapine-induced hypersensitivity [53,54]. In addition to HLA associations, the immunological response to nevirapine is CD4+ cell count dependent [51]. Interestingly, lower pre-treatment CD4 T-cell counts are protective against the development of hypersensitivity which accordingly leads to more severe reactions in HIV-negative individuals who receive prophylactic treatment with nevirapine [55]. These contradictory findings highlight the need for further research in the area and clearly indicate that clinical utility for pre-treatment genetic testing for nevirapine hypersensitivity has not yet been demonstrated.

## 6. Anticonvulsants and HLA-B*1502

The strongest association (OR > 1,000) between HLA alleles and drug-induced hypersensitivity has been detected for carbamazepine (CBZ)-induced SJS/TEN in the Han Chinese population [35,36,56]. The association was later confirmed in several other Asian populations including Thai [37,57]), Malay [58] and Indian [59], but not in Caucasians [34,38,60]. Interestingly, the frequency of SJS/TEN is higher in Asian populations than in Caucasians, as is the frequency of the B*1502 allele (http://www.allelefrequencies.net/) which provides circumstantial evidence for its potential functional relevance.

Apart from being ethnicity-specific, the genetic marker for CBZ induced hypersensitivity is phenotype specific. HLA-B*1502 is only valid for SJS/TEN, but not for maculopapular exanthema or DRESS (drug reaction with systemic symptoms) (Table 4) [36]. Maculopapular exanthema has been associated with HLA-A*3101 and DRESS with the polymorphisms in the motilin gene in the Han Chinese population [36]. In Caucasians, DRESS has been associated with the ancestral haplotype HLA-B8.1 [61].

Patients who develop hypersensitivity to one aromatic antiepileptic drug (AED) may also show cross-reactivity to structurally related AEDs [62]. Several case reports have shown that the HLA-B*1502 allele may be important in patients with phenytoin, lamotrigine and oxcarbazepine-induced SJS [37,56,63]. These findings however need to be validated in a larger number of ethnically diverse populations. In Caucasians, lamotrigine-induced DRESS has been associated with the HLA alleles, B*5801 and A*6801 [64], although the association was relatively weak and unlikely to be useful clinically.

## 7. Drug-Induced Liver Injury (DILI)

DILI can take many forms, which in general have been thought to be due to metabolic and immunologically mediated mechanisms. Recent investigations with a number of drugs associated with liver injury where the predominant clinical picture was either hepatitic (a rise in transaminases), cholestatic (a predominant rise in alkaline phosphatase and gamma-glutamyl transferase), or a mixed phenotype, have shown a very strong association with HLA alleles indicating an immune aetiology even though the clinical picture did not show any other phenomena usually regarded as depicting an immune aetiology, e.g. fever, eosinophilia, and short time to onset of liver injury. These are considered below.

**Flucloxacillin**: A penicillinase resistant beta-lactam antibiotic used in the treatment of *Staphylococcus aureus* infections that can cause cholestatic hepatitis in 8.5 per 100,000 exposed individuals [65]. In a recent study by Daly *et al.* [66] which utilised a genome wide approach, a striking association between the rs2395029, the tag SNP for HLA-B*5701 in the HCP gene, and flucloxacillin-induced hepatotoxicity was detected. HLA-B genotyping found that all patients with the rs2395029 polymorphism were also positive for HLA-B*5701. As the positive predictive value (PPV) of the test depends on the prevalence of the disorder, in this example, there is a very low PPV indicating that approximately only 1 in 500-1,000 individuals positive for HLA-B*5701 would develop liver damage if exposed to flucloxacillin [66]. Therefore, pre-treatment genetic testing is unlikely to be clinically utilised. However, the use of HLA-B*5701 to confirm the diagnosis of flucloxacillin-induced cholestatic hepatitis should be considered where there are competing aetiologies.

**Amoxicillin-clavulanate (co-amoxiclav)** is a combination antibiotic efficient against amoxicillin-resistant bacteria that produce β-lactamase. It can cause hepatic injury, predominantly cholestatic, which has also been linked to HLA alleles. Patients who are carriers of HLA-DRB1*1501 alleles are at risk of developing toxicity [67,68]. These reports have been based on a small number of patients who were recruited from single centres. A UK-wide multicentre study conducted recently was able to confirm the association between co-amoxiclav hepatotoxicity and HLA-DRB1*1501 (OR 2.59: 95% CI 1.44-4.68), but in addition found a protective effect of HLA-DRB1*07 family of alleles in the same cohort [69]. A large multinational GWAS study led by the Serious Adverse Events Consortium which has investigated 200 patients with co-amoxiclav hepatotoxicity is currently underway.

**Lumiracoxib:** HLA-DRB1*1501 allele has also recently been identified to be associated with lumiracoxib-related liver injury in a recent genome-wide study [70]. Lumiracoxib is a selective cyclooxygenase-2 inhibitor that is efficacious in the symptomatic treatment of osteoarthritis and acute pain [71]. The drug was withdrawn from the market because of concerns of hepatotoxicity at a rate of 6.39 per 100,000 users [72]. Singer *et al.* carried out a GWAS on prospectively collected samples as a part of phase III clinical safety study which involved 18,000 patients with osteoarthritis [70]. The authors reported that the HLA haplotype HLA-DRB1*1501-DQB1*0602-DRB5*0101-DQA1*0102 was associated with lumiracoxib toxicity. The low positive predictive value of this haplotype indicates that there are other factors that can trigger hepatotoxicity in patients. Interestingly however, the sensitivity of one allele, DQA1*0102 seems to correlate well with the severity of liver injury as measured by an elevation of transaminases. The sensitivity was 73.6% for patients with ALT elevations to >3XULN, but increased to 84% for those with ALT at >5XULN, 91% for >8XULN and reached100% for patients with ALT > 20XULN [70].

**Lapatinib:** Researchers from GlaxoSmithKline have conducted a pharmacogenetic investigation of lapatinib-induced transaminase elevation in women with advanced or metastatic breast cancer. They performed 1 million SNP GWAS in two studies, an exploratory and a confirmatory study, to show an association with DQA1*0102 with an odds ratio of 9.2 and positive and negative predictive values of 0.18 and 0.97, respectively (http://www.genomeweb.com/dxpgx/asco-2010-pgx-abstract-highlights/).

**Ximelagatran** is an anticoagulant that was developed as a replacement for warfarin because of its problematic dosing. However, ximelagatran had to be withdrawn in 2006 following reports of hepatotoxicity in clinical trials [73]. A GWAS in 74 patients with ximelagatran-induced transaminitis and 169 controls using a retrospective case-control design [74]. In contrast to the study on co-amoxiclav hepatotoxicity mentioned earlier where DRB1*0701 was found to be protective, in case of ximelagatran, HLA Class II DRB1*0701 allele conferred an increased risk of toxicity [74]. This finding however, has not been replicated in an independent small cohort of patients consisting of 10 cases and 16 treated controls [74].

Taken together, the above results of HLA allele associations and DILI raise several issues: (1) HLA alleles as well as their haplotypes seem to be important in drug-induced liver toxicity; (2) associations with common alleles indicate that these alleles are neither necessary nor sufficient to cause toxicity and that there is a need for further studies; (3) causality needs to be established in functional mechanistic studies as these associations could be a reflection of strong LD in this genomic region; and (4) there is overlap in the HLA associations between structurally and therapeutically different drugs and the risk of DILI. For example, HLA-DRB1*1501 is associated with hepatotoxicity associated with both lumiracoxib and co-amoxiclav, HLA-B*5701 is associated with both abacavir hypersensitivity and flucloxacillin cholestatic hepatitis, and HLA-DQA1*0102 is associated with both lumiracoxib and lapatinib transaminitis. Whether the overlap is co-incidental or reflects a common mechanism is not clear at present – if the latter, then it might be possible in the future to genotype individuals for a number of alleles to prevent different types of immune-mediated adverse reactions such as DILI.

## 8. Clinical Utility of Pre-Treatment (Predictive or Diagnostic) Genetic Testing

The predictive value of HLA-B*5701 testing for preventing abacavir hypersensitivity has been confirmed in a prospective clinical trial and is already being implemented in many high- to medium-income countries. Cost-effectivness has also been demonstrated [23]. Recently, Chen and colleagues at Academia Sinica in Taiwan conducted a prospective clinical trial of nearly 5,000 patients who were prescribed carbamazepine to assess the cost-effectivness of HLA-B*1502 pre-treatment testing. They demonstrated that genotyping for HLA-B*1502 could reduce the occurrence of SJS/TEN and that the healthcare system could save millions if CBZ was prescribed only to those individuals who are B*1502 negative (www.genomeweb.com). However, it is important to keep in mind that clinical observation and close monitoring of patients should still be conducted even if they are negative for the HLA alleles implicated in hypersensitivity.

Robust genetic markers to predict susceptibility to liver injury are still lacking. However, the HLA allelic associations may also be clinically beneficial through use as (a) tests of exclusion, as has been proposed for the re-introduction of lumiracoxib (http://www.genomeweb.com/dxpgx/novartis-adds-companion-dx-lumiracoxib-resubmission-europe); and (b) diagnostic aids, as has been suggested for flucloxacillin-induced cholestatic hepatitis [66].

## 9. Conclusions

Huge advances in genotyping technology in recent years has not yet been translated into clinical utility, but this is probably only a matter of time. In order to be in a position to take advantage of these advances, the international scientific community needs to continue in, and accelerate, joint efforts to collect and store clinically well characterized biological samples which could be used to identify or confirm DNA variants useful in predicting adverse drug reactions. In addition, research into the functional mechanisms of genetic associations found with hypersensitivity reactions should be considered in order to identify causal variants, strengthen the basis of associations, and ultimately allow for the development of diagnostic tests, and in the future, lessons for safer drug design.

## Figures and Tables

**Table 1:** Clinical classification of delayed-type hypersensitivity reactions (adapted from Posadas and Pichler; Torres *et al.*) [75,76].

**Reaction**	**Cell type, Antibody**	**Immune mechanism**	**Clinical manifestation**
Delayed-type hypersensitivity	Drug-specific T lymphocytes		
Type IVa	Th1	Monocyte/macrophage activation; secretion of IFN-γ and TNF-α; perforin and granzyme B secretion	Contact dermatitis, bullous skin rash
Type IVb	Th2	Secretion of IL-4, IL-13 and IL-5 cytokines; B-cell production of IgE	Eosinophil-rich maculopapular exanthema, bullous skin rash
Type IVc	Cytotoxic T cells	Activated CD8^+^ T cells kill keratinocytes	Maculopapular and bullous skin rash, contact dermatitis, hepatitis, nephritis, SJS, TEN
Type IVd	T cells	Recruitment and activation of neutrophils via CXCL8 release and apoptosis prevention via GM-CSF release	Sterile neutrophilic inflammations-AGEP

**Legend:** AGEP - acute generalized exanthematous pustulosis; SJS - Stevens-Johnson syndrome; TEN - toxic epidermal necrolysis; Th1- T-helper type 1; Th2- T-helper type 2; CXCL8 - neutrophil chemotactic and activating factor 8 (IL-8); GM-CSF - granulocyte-macrophage colony-stimulating factor.

**Table 2:** Viral infections and drug hypersensitivity.

**Viral infection**	**Drug-reaction**	**References replace years with ref numbers**
Epstein-Barr virus (EBV) Infectious mononucleosis	Ampicillin induced exanthema	Shiohara 2007; Gonzales-Delgado 2006; Renn 2002 [25,27,28]
Human herpes virus 6 (HHV-6)	DRESS induced by CBZ, teicoplanin, vancomycin	Peyriere 2006; Watanabe 2009; Tamagawa-Mineoka 2007; Descamps 2001; Aihara 2003; Kano 2004;Calligaris 2009 [31,101-106]
Cytomegalovirus (CMV)	Tribenoside-induced hypersensitivity syndrome	Hashizume 2005 [107]
Human immunodeficiency virus (HIV)	SMX- induced hypersensitivity	Bigby JAMA 1986; Gordin 1984 [108,109]
Human immunodeficiency virus (HIV) and reactivation of EBV or CMV	Trimethoprimsulphamethoxazole-induced hypersensitivity or SJS/TEN	Smith 1997 [33]

**Table 3:** An overview of HLA allele associations with drug-induced hypersensitivity in different populations.

**Drug**	**Class of drug**	**HLA-allele**	**Population**	**Reference**
**SJS/TEN**				
Allopurinol	anti uric acid	B*5801	Han Chinese	Hung 2005 [77]
			Thai	Tasseneeyakul 2009 [78]
			Japanese	Kaniwa 2008 [79]
			Malay	Ding 2010 [58]
Carbamazepine	antiepileptic	B*1502	Han Chinese	Chung 2004; Hung 2006; Man 2007 [35,36,56]
			Thai	Locharernkul 2008; Tasseneeyakul 2010 [37,57]
			Malay	Ding 2010 [58]
			Indian	Mehta 2009 [59]
Phenytoin	antiepileptic	B*1502	Han Chinese	Man 2007 [56]
			Thai	Locharernkul 2008 [37]
Oxicam	NSAID	A2, B12	Caucasians	Roujeau 1987 [80]
Sulphamethoxazole	antibiotic	A29, B12, DR7	Caucasians	Roujeau 1986 [81]
**Hypersensitivity syndrome (DIHS or DRESS)**				
Abacavir	antiretroviral	B*5701	Caucasians	Mallal 2002; Hetherington 2002; Martin 2004; Huhges 2004; Mallal 2008 [14,22,24,82]
			African Americans	Hughes 2004b; Saag 2008 [39,83]
Aminopenicillins	antibiotic	A2, Drw52	Caucasians	Romano 1998 [84]
Nevirapine	antiretroviral	DRB1*01	Caucasian-Australian	Martin 2005 [51]
		DRB1*01	Caucasian-French	Vitezica 2008 [52]
		Cw8,B14	Caucasian-Italian	Littera 2006 [50]
		Cw8	Japanese	Gatanaga 2007 [49]
		B*3505	Thai	Chantarangsu 2009 [53]
		Cw4	Thai	Likanonsakul 2009 [54]
Aspirin	NSAIDS	DRB1*1302, DQB1*0609		Kim 2005; Palikhe 2008 [85,86]
	NSAIDS	DR11		Quiralte 1999 [87]
Iodine contrast media		DR	Caucasians - Spanish	Torres 2008 [88]
Paraphenylenediamine	Hair dye	DP	Caucasians-German	Sieben 2002 [89]
Gold sodium thiomalate	Treatment of rheumatoid arthritis	DR5	Caucasians - Spanish	Rodriguez-Pérez 1994 [90]
Lamotrigine	antiepileptic	B*5801, A*6801	Caucasians	Kazeem 2009 [64]
Trichloroethylene	Industrial solvent, dry cleaning	B*1301	Japanese	Li 2007; Watanabe, 2010 [91,92]
**Fixed drug eruptions**				
Co-trimoxazole	antibiotic	A30 B13 Cw6	Caucasians-Turkish	Ozkaya-Bayazit 2001 [93]
Feprazone	analgesic	B22		Pellicano 1997 [94]
**Drug-induced hypersensitivity - nephritis**				
Minocycline	anti-acne agent	DR	Japanese	Joh 1990 [95]
Penicillin	antibiotic	DR	Japanese	Joh 1990 [95]
NSAIDs		DR	Japanese	Joh 1990 [95]
**Drug-induced hypersensitivity - liver toxicity**				
Flucloxacillin	antibiotic	B*5701	Caucasian,	Daly 2009 [66]
Ximelagatran	antiplatelet	DRB1*0701		Kindmark, 2008 [74]
	agent	DQA1*02		
Lumiracoxib	Antiarthritic	DRB1*1501		Singer 2010 [70]
	drug	DQA1*0102		
Co-amoxiclav	antibiotic	DRB1*1501		Hautekeete, 1999; O'Donohue 2000 [67,68]
Lapatinib	anticancer	DQA1*0102		web
Ticlopidine	antiplatelet	A*3303		Hirata 2008 [96]
Diclofenac	NSAID	DRB1*13		Daly 2009 [97]
Clometacin	analgesic	B8		Pariente 1989 [98]

**Table 4:** Clinical phenotype and symptoms of drug hypersensitivity (Adapted from ENDA (European Network for Drug Allergy) questionnaire) [99,110,111].

**Cutaneous symptoms**	**Gastrointestinal and respiratory symptoms**	**Cardiovascular symptoms**	**Associated symptoms**
Maculopapular exanthema	Nausea/ Vomiting	Tachycardia	Fever
Macular exanthema	Diarrhoea	Hypotension	Liver involvement
Urticarious exanthema	GI cramps	Collapse	Kidney involvement
Acute generalised exanthemous pustulosis (AGEP)	Cough	Arrhythmia Myocarditis	Lymphadenopathy
Erithema exudativum multiforme	Dyspnea		Malaise
Bullous exanthema	Wheezing/Bronchospasm		Pain/burning
Stevens Johnson syndrome/Toxic epidermal necrolysis (Morbus Lyell)	Rhinitis		Oedema
Fixed drug exanthema	Sneezing		Arthralgia/Myalgia
Purpura	Nasal obstruction		**Biochemical tests**
Contact dermatitis			Eosinophilia
Urticaria vascularis			Atypical lymphocytes
Pruritus Angioedema			
